# Histone content increases in differentiating embryonic stem cells

**DOI:** 10.3389/fphys.2014.00330

**Published:** 2014-08-28

**Authors:** Theodoros Karnavas, Luisa Pintonello, Alessandra Agresti, Marco E. Bianchi

**Affiliations:** ^1^Chromatin Dynamics Unit, San Raffaele University and Research InstituteMilan, Italy; ^2^HMGBiotech SrlMilan, Italy; ^3^Core Facility for Conditional Mutagenesis, San Raffaele Research InstituteMilan, Italy; ^4^Center for Translational Genomics, San Raffaele Research InstituteMilan, Italy

**Keywords:** embryonic stem cells (ESCs), differentiation, epigenetics, development, Histones

## Abstract

Mouse Embryonic Stem Cells (ESCs) are pluripotent mammalian cells derived from the Inner Cell Mass (ICM) of mouse blastocysts, which give rise to all three embryonic germ layers both *in vivo* and *in vitro*. Mouse ESCs have a distinct epigenetic landscape and a more decondensed chromatin compared to differentiated cells. Numerous studies have shown that distinct histone modifications in ESCs serve as hallmarks of pluripotency. However, so far it is still unknown whether the total histone content (as opposed to histone modifications) remains the same in cells of different developmental stage and differentiation capacity. In this work we show that total histone content differs between pluripotent and differentiated cells. *In vitro* spontaneous differentiation from ESCs to Embryoid Bodies (EBs) and directed differentiation toward neuronal and endodermal cells entails an increase in histone content. Primary MEFs also contain more histones than ESCs. We suggest that the difference in histone content is an additional hallmark of pluripotency, in addition to and besides histone modifications.

## Introduction

Different histone modifications are known to mark chromatin in cells of different developmental stage. Though, very little is known about possible differences in histone content among cell types. Here we investigated whether total histone content differs significantly among pluripotent and differentiated cells.

Embryonic Stem Cells (ESCs) are pluripotent cell lines derived from Inner Cell Mass (ICM) of blastocysts (E3.5 in the mouse) (Evans and Kaufman, 1981). ESCs have the ability to self-renew indefinitely *in vitro* and to contribute to all embryonic tissues in chimeras and ultimately to all different cell types in adults. ESCs can also differentiate *in vitro* into different cell types (Keller, 2005; Murry and Keller, 2008). Once differentiation is initiated, lineage specification occurs by the implementation of genome expression programmes that give each cell type a unique transcriptional profile. These functional properties place opposing constraints on the genome of ESCs: self-renewal requires that ESC maintain a cell memory that specifies pluripotency, whereas the genome must be in a highly plastic state so as to enter distinct differentiation pathways.

In ESCs the majority of chromatin appears decondensed and the genome is transcriptionally hyperactive, whereas large sections of the genome undergo progressive silencing as cells differentiate (Efroni et al., 2008). Nucleosome remodeling and histone modifications, mostly in histone tails, have been suggested to play a significant role in these processes (Chen and Dent, 2014). Differentiation correlates with an increase in H3K9me3, an epigenetic marker for silenced heterochromatin, and a decrease in H3 and H4 acetylation, which is associated with transcriptionally active euchromatin. However, very little is known about possible differences in total histone content. For a long time the amount of histones in a nucleus has been tacitly considered a fixed parameter, essentially corresponding to the length of DNA to be packaged into nucleosomes. Recently, a decrease in histone content and nucleosome number during aging has been described by two different groups (Feser et al., 2010; O'Sullivan et al., 2010). Our group showed (Celona et al., 2011) that histone content is reduced in the absence of the chromatin protein HMGB1. Cells with reduced histones, as expected, have a more accessible chromatin and increased transcription.

In this study we show that ESCs contain about 30% less histones than differentiated cells deriving from them either spontaneously (Embryoid Bodies, EBs) or by inducing specific differentiation programs to neuronal and endodermal cells. ESCs also contain less histones than primary MEFs directly derived from E14.5 embryos. We suggest that the difference in histone content between ESCs and differentiated cells may represent a new layer of control of the epigenome.

## Materials and methods

### Cell culture

**Figure 1:**
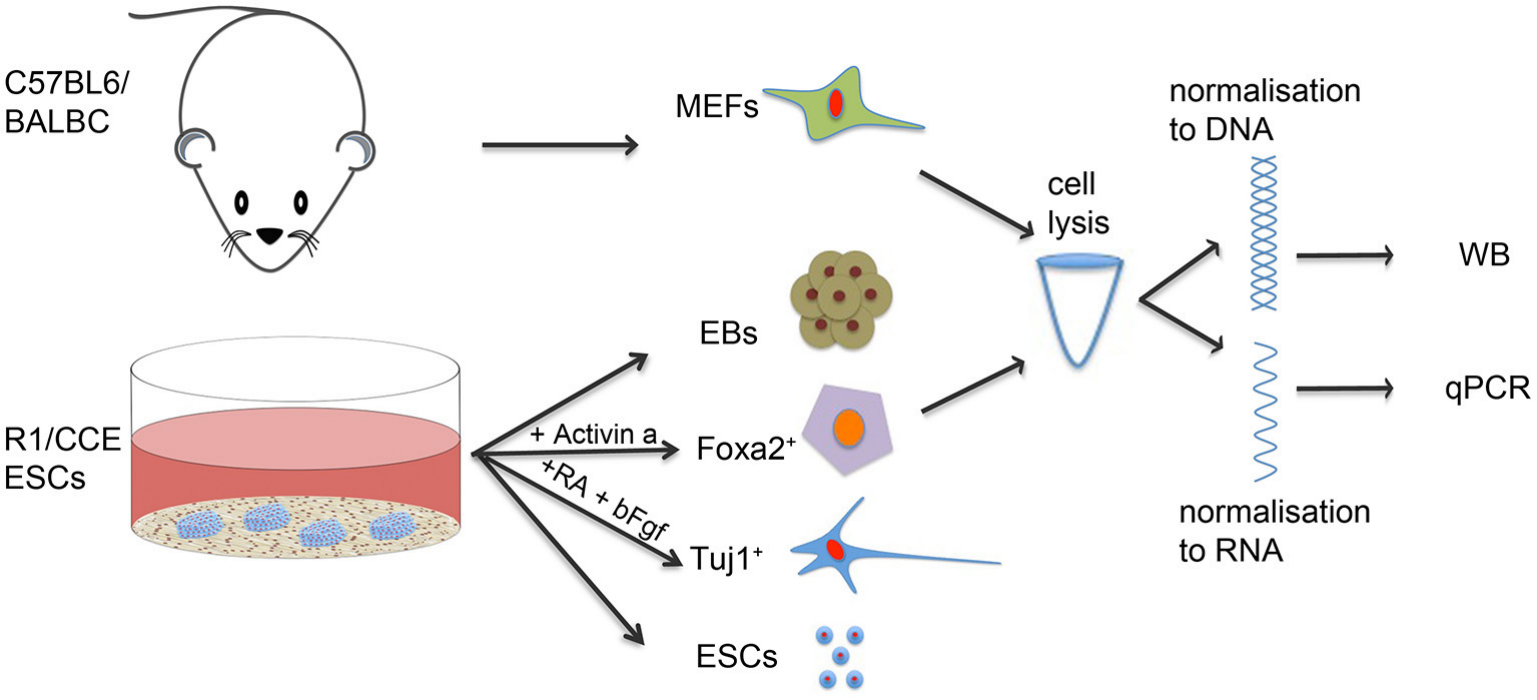
**Experimental outline**. R1 and CCE ES cells (ESCs) were cultured under ESC culture conditions or differentiated either spontaneously to Embryoid Bodies (EBs) or specifically to differentiated cells expressing neuronal (TUJ1) and endodermal (FOXA2) markers. Mouse Embryonic Fibroblasts (MEFs) from C57/BL6 and BALB/C background were also used as examples of differentiated cells. Cell samples were lysed and subjected to qPCR and western blot analysis to assess total histone H3 mRNA and protein content.

The experimental outline of the work is shown in Figure 1. ESCs from 2 different backgrounds, CCE and R1, were cultured on a feeder layer of Mouse Embryonic Fibroblasts (MEFs) that had been inactivated with mitomycin C, in DMEM containing 15% FCS plus Leukemia Inhibitory Factor (LIF, 10^3^ u/ml), non-essential amino acids (1 mM), sodium pyruvate (1 mM), β-mercaptoethanol (50 μM), penicillin–streptomycin (100 μg/ml), L-glutamine (2 mM), and 5% glucose. Medium was changed every single day. ESCs cultured for 2 sequential passages; to separate them from MEFs, trypsinized cells were plated in tissue culture dishes for 30 min: MEFs adhere to the plates while ESCs remain unattached. ESCs were collected and lysed to be processed for mRNA and protein analysis.

Alternatively, ESCs were plated at a density of 5 × 10^4^ cells/ml in dishes without coating and let to grow in ESC medium without LIF as aggregates in suspension—EBs. Medium was changed for the first time after 48 h and then every other day. EBs were collected at days 2, 4, and 8 and lysed to be processed for mRNA and protein analysis.

ESCs grown for 2 sequential passages in ESC culture conditions as described above were also differentiated toward neuronal (NE) and endodermal (E) cells (Li et al., 1998; Kubo et al., 2004). For NE, ESCs were deprived of LIF for 8 days, and during the last 4 days all-trans retinoic acid (ATRA, 1 mM) was added. After day 8 EBs were trypsinized, dissociated to single cells and plated at 37°C at a density of 2.5 × 10^4^ cells/cm^2^ in tissue culture multiwells precoated with poly-D-lysine (10 μg/ml) for 2 h, and then in multiwells precoated with laminin (10 μg/ml) for 2 h. Cells were then cultured in neural specific medium containing DMEM-F12/Hams, 0.1 mM β-mercaptoethanol, 2 mM L-glutamine, 100 μg/ml penicillin-streptomycin, 0.6% glucose, 20 ng/ml bFGF, 25 μg/ml insulin, 5.2 ng/ml sodium selenite, 9.6 ng/ml putrescine, 6.3 ng/ml progesterone, and 100 μg/ml apo-transferrin; the medium was changed on day 2. At day 4 cells were transferred for 4 more days for further neuronal differentiation to medium consisting of 1:1 mixture of neural specific medium without bFGF and neurobasal medium containing 2 mM L-glutamine and B27 supplement 50× (GIBCO); the medium was changed on day 2. Cells were then collected to be subjected to qPCR, WB, and immunofluorescence analysis.

For differentiation toward endodermal lineage, ESCs were placed in dishes at a density of 8 × 10^4^ cells/cm^2^ to grow as aggregates for 2.5 days without medium change, in IMDM (+L-glutamine) containing 15% FCS, 10 μg/ml gentamycin, 0.5 mM ascorbic acid, 0.1 mM β-mercaptoethanol, and 200 μg/ml apo-transferrin. The third day aggregates were collected by gravity and replated in IMDM (+L-glutamine), gentamycin (10 μg/ml), ascorbic acid (0.5 mM), β-mercaptoethanol (0.1 mM), 15% serum replacement and A-activin (10 ng/ml). Aggregates remained for 3.5 days in this medium with an intermediate medium change. After 3.5 days in culture A-activin was withdrawn and aggregates were plated in gelatin precoated multiwells for 4 days in IMDM (+L-glutamine), gentamycin (10 μg/ml), ascorbic acid (0.5 mM), β-mercaptoethanol (0.1 mM), 15% FCS, and dexamethasone (0.1 μM).

Mouse embryonic fibroblasts (MEFs) isolated from strains BALB/C, C57/BL6, and SV129 were plated in high-glucose DMEM containing 10% FCS, non-essential amino acids (1 mM), sodium pyruvate (1 mM), β–mercaptoethanol (50 μM), Penicillin–Streptomycin (100 μg/ml), L-glutamine (2 mM). MEFs were cultured for four passages at most.

### Total RNA isolation, reverse transcription, and qPCR

Total RNA was isolated using the GE Healthcare RNAspin Mini kit, and quantified with Nanodrop. Reverse transcription was performed using 200 units SuperScript II reverse transcriptase (Invitrogen), 1 μg total RNA, reverse transcription buffer, 10 mM dNTPs, 50 ng random primers (hexamers), and 10 mM DTT. qPCR was performed on a LightCycler using the LightCycler 480 DNA SYBR Green 1 Master kit (Roche), and primers for H3 (fwd: 5′-taccagaagtcgaccgagctg-3′ and rev: 5′-aggttggtgtcctcaaacaga-3′) and for 28S rRNA (fwd: 5′-gcgacctcagatcagacgtgg-3′ and rev: 5′-cttaacggtttcacgccctc-3′). The primers for H3 were designed to amplify transcripts from all H3 genes.

### Western blots

Cells were lysed at room temperature either in SDS-PAGE loading buffer or in RIPA buffer and processed for H3 protein and SOX2 protein respectively. DNA was quantified in the lysates using the Quant-iT PicoGreen dsDNA Assay kit (Invitrogen). Samples containing defined amounts of DNA were loaded in the lanes of 15% SDS-PAGE gels. Each sample was run in three different concentrations, in two technical replicates each. For SOX2, lysates in RIPA buffer were quantified for total protein amount with the Bradford method; GAPDH was used as a loading control. All antibodies (GAPDH Abcam 8245, SOX2 Abcam 59776, H3 Abcam 1791, H4 Abcam 7311, H2A Millipore 07-146, H2B Millipore 07-31) were used according to the manufacturer's instructions. Filters were scanned with Typhoon FLA 9000 (GE Healthcare) and protein bands were quantified using the ImageJ64 band intensity program (Schneider et al., 2012). Statistical means and standard deviations for technical replicates were estimated and the values were finally corresponded to the respective amount of DNA loaded in order to design the standard curve for serial dilutions loaded for one reference sample.

### Immunofluorescence

Neural and endodermal cells were fixed in freshly prepared ice-cold 5% PFA diluted in 0.12 M phosphate buffer, pH 7.4 (PB) for 25 min at 4°C, blocked for 1 h at RT using blocking buffer (PB containing 0.15% glycine, 0.1% Triton-X, and 2 mg/ml BSA). FOXA2 and TUJ1 primary antibodies (Ab) were used according to the manufacturer's instructions in blocking buffer O/N at 4°C. Cells were washed with PBT (PBS containing 0.1% Triton-X) for 10 min at RT for three times, then incubated with secondary antibody (goat anti-rabbit conjugated with AlexaFluor 488, from Alexa). After further washing, cells were incubated DAPI mounting medium for 20 min at RT and visualized.

### Statistics

For both qPCR analysis and western blot analysis values of technical replicates from individual samples (biological replicates) coming from different experiments were subjected to non-parametric statistical tests. Mann–Whitney tests were used for the comparison of two biological replicates, and Kruskal-Wallis tests for the comparison of more than two biological replicates. In all cases *p*-values < 0.05 were considered statistically significant.

## Results

### ESCs express less H3 mRNA and contain less H3 protein compared to primary MEFs

Core histones are present in stoichiometric amounts, even in cell with low content, such as aging cells or cells lacking the HMGB1 chromatin protein (Feser et al., 2010; O'Sullivan et al., 2010; Celona et al., 2011). Thus, measuring histone H3 is representative of all core histones.

**Figure 2:**
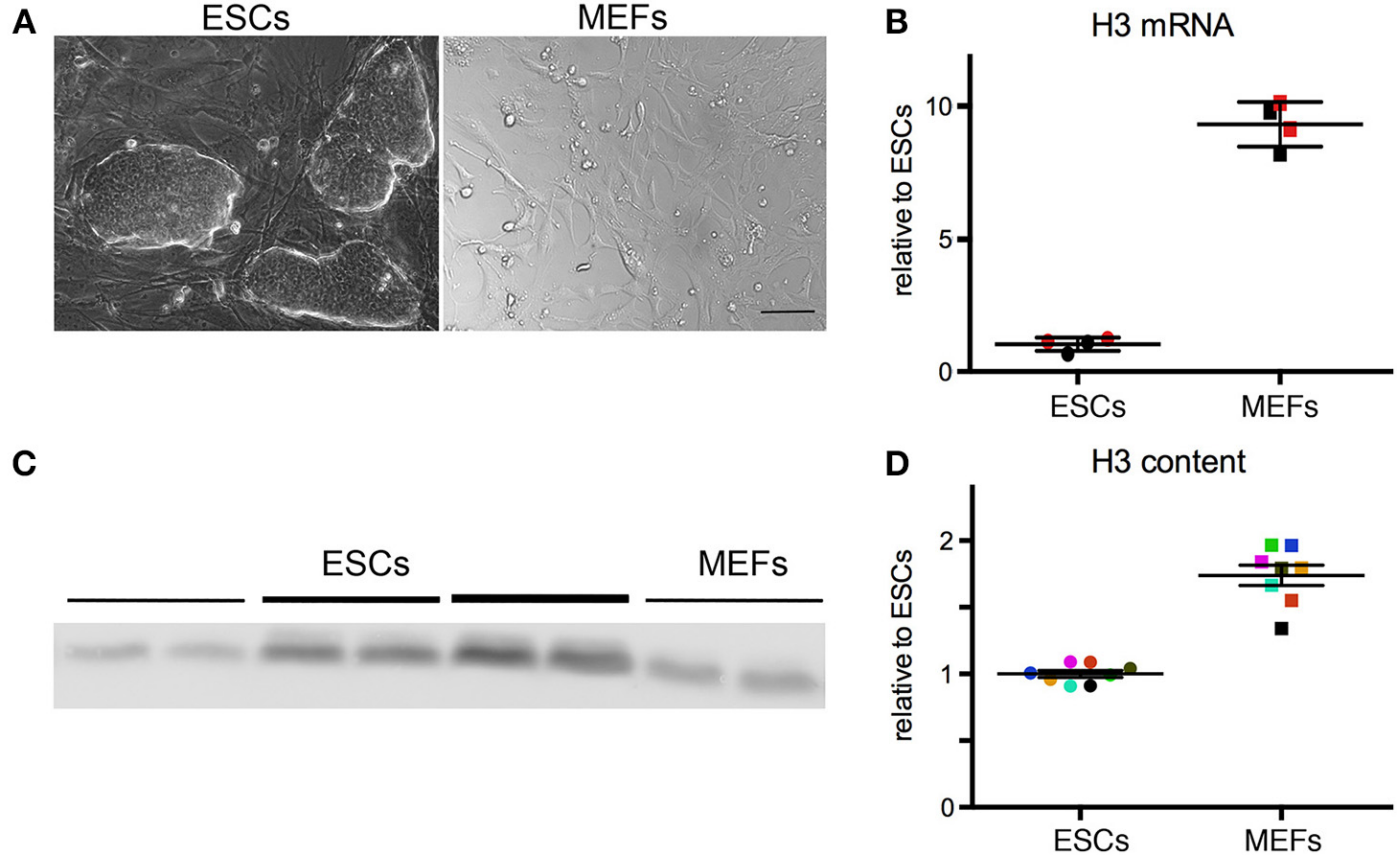
**(A)** R1 and CCE ESCs were cultured under ES-like conditions, and C57/BL6 and BALB/C MEFs isolated from E14.5 embryos were cultured under standard conditions. Scale bar 50 μm. **(B)** ESCs express less H3 mRNA than MEFs. Each dot represents a different biological replicate. Color codes identify samples analyzed in the same batch the same day; different colors represent samples analyzed in different days. The mean and standard deviation are indicated by horizontal lines. The groups are statistically different (Mann-Whitney test, *p* = 0.029). **(C)** Western blot for H3 protein. Lines of different thickness represent loading amounts: equivalent to 100, 200, 300 ng of DNA. **(D)** MEFs contain more H3 protein than ESCs. The different samples are normalized for H3/DNA ratio. The values are normalized relative to the mean of the ESC group, which is set to 1. Each dot represents a different biological replicate. Color codes identify samples analyzed in the same batch the same day; different colors represent samples analyzed in different days. The groups are statistically different (Mann-Whitney test, *p* = 0.0002).

The experimental outline of the work is shown in Figure 1. As a first step, we compared the expression of H3 histone in ESCs and primary MEFs. ESCs of different backgrounds (CCE and R1) were cultured under typical ESC tissue culture conditions: DMEM containing 15% FCS and the pluripotency sustaining factor LIF (Figure 2A). MEFs were isolated from E14.5 embryos (Celona et al., 2011) of various strains (BALB/C, C57/BL6, and SV129) and cultured *ex vivo* under standard culture conditions (Figure 2A) until passage 3. ESCs and MEFs were lysed and subjected to total RNA purification and subsequent qPCR analysis; each sample is a biological replicate. Total RNA was retrotranscribed using random primers, which ensures that every RNA molecule in the sample will be converted to cDNA; this is necessary because most histone mRNAs lack a polyA tail. qPCR was then performed using primers that amplify all transcripts for histone H3 and ribosomal RNA 28S, which was used as a normalizer. This analysis (Figure 2B) indicated that the amount of H3 mRNA relative to 28S is approximately 8-fold larger in MEFs relative to ESCs (*p* = 0.029, Mann–Whitney test). Our measure does not reflect directly the number of H3 mRNA molecules per cell, but only the amount relative to 28S rRNA; since ribosomes are less abundant in ESCs than in differentiated cells (Sampath et al., 2008), the H3/28S ratio can only overestimate H3 in ESC cells and underestimate the increase in H3 transcripts during differentiation.

Next, we measured the amount of H3 protein relative to total DNA content. This measure is proportional to the density of H3 histone on DNA, and thus to the number of nucleosomes per cell, as over 99% of histones are deposited into nucleosomes (van Holde, 1988). One aliquot from each cell sample was lysed in SDS-PAGE loading buffer, and DNA was quantified before loading on a SDS-PAGE gel. Protein samples were loaded in serial dilutions, and thus each biological sample was measured in technical triplicates; an example of the Western blots is shown in Figure 2C. Overall, this analysis indicated that MEFs contain on average 1.7-fold more H3 histone than ESCs (Figure 2D; *p* = 0.0002; Mann–Whitney test).

To exclude the possibility that the observed difference in H3 content may be due to different genetic background, MEFs and ESCs from the same strain (SV129) were processed for H3 content analysis, again confirming that MEFs contain more H3 histone than ESCs (data not shown).

### ESCs express less H3 mRNA and contain less H3 histone compared to differentiating embryoid bodies

**Figure 3:**
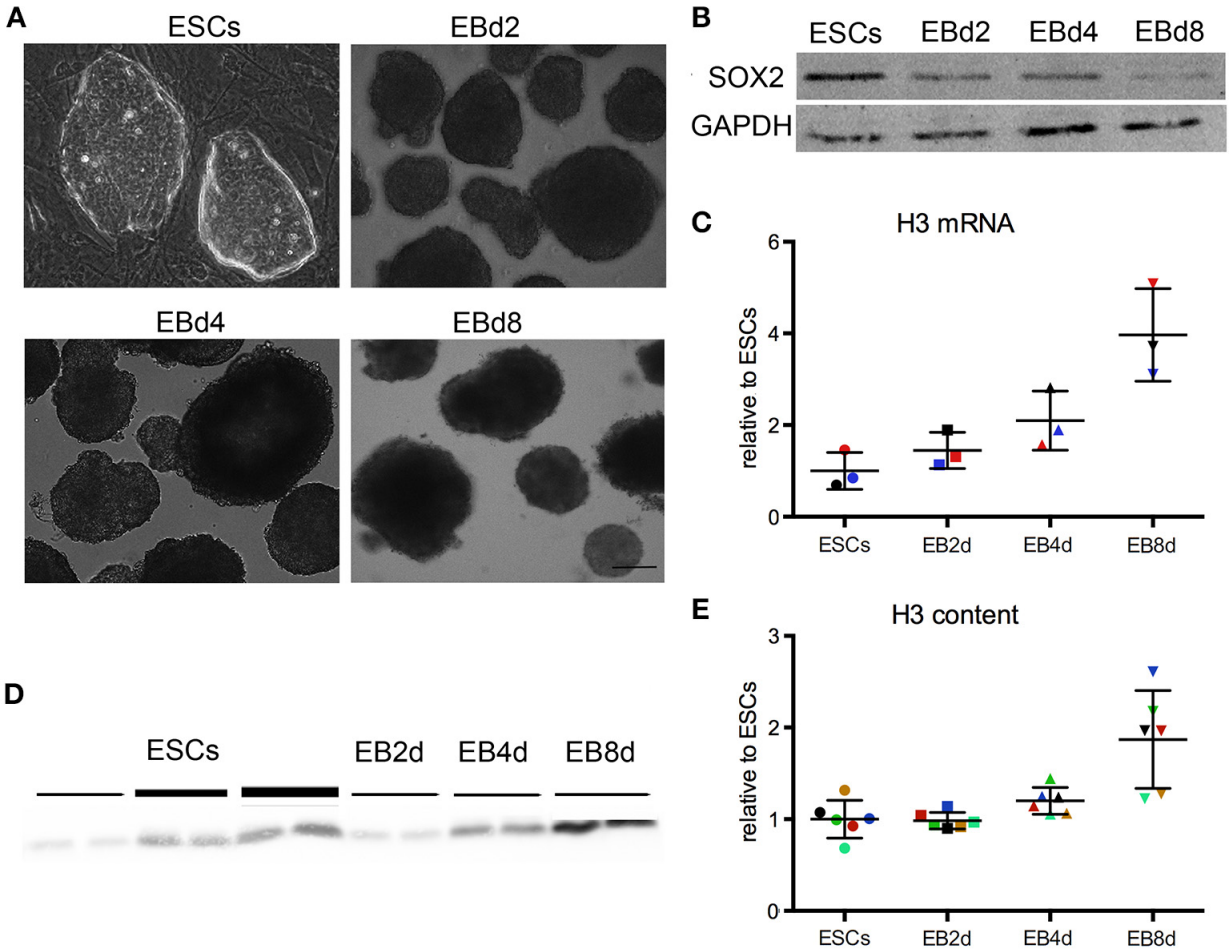
**(A)** CCE and R1 ESCs were cultured under ES like conditions and differentiated spontaneously to Embryoid Bodies (EB) for 2, 4, and 8 days. We found no statistical difference between R1 and CCE cells, or between EBs derived from the different ESC lines. Scale bar 50 μm. **(B)** Western blot for SOX2 protein, which gradually decreases in EBs. **(C)** ESCs express less H3 mRNA. Each dot represents a different biological replicate. Color codes identify samples analyzed in the same batch the same day; different colors represent samples analyzed in different days. The mean and standard deviation are indicated by horizontal lines. The groups are statistically different (Kruskal–Wallis test, *p* = 0.0028). **(D)** Western blot for total H3 protein. Lines of different thickness represent loading amounts: equivalent to 100, 200, 300 ng of DNA. **(E)** ESCs contain less H3 protein. The H3 protein content was normalized to DNA amount. The values were further normalized relative to the mean of the ESC group, which is set to 1. Each dot represents a different biological replicate. Color codes identify samples analyzed in the same batch the same day; different colors represent samples analyzed in different days. The groups are statistically different (Kruskal–Wallis test, *p* = 0.0021).

The difference in H3 histone content between ESCs and MEFs suggests that histone content may be larger in differentiated cells compared to pluripotent ones. To test this notion directly, we differentiated *in vitro* ESCs of different backgrounds (CCE and R1) into EBs. ESCs, initially cultured using DMEM medium plus 15% FCS and the pluripotency sustaining factor LIF, were let to differentiate spontaneously to EBs in the absence of LIF for 8 days (Figure 3A). EBs are mixed populations of differentiating cells, which as differentiation proceeds lose gradually their pluripotent and multipotent component. Correspondingly, the pluripotent protein SOX2 gradually declined in EBs over time (Figure 3B).

ESCs and EBs from days 2, 4, and 8 were lysed and subjected to total RNA purification and subsequent qPCR analysis; each sample is a biological replicate and was analyzed as explained before. The amount of H3 mRNA relative to 28S increases in differentiating EBs relative to ESCs (*p* = 0.0028, Kruskal-Wallis test), and becomes 4-fold larger on average in 8-day EBs (Figure 3C). Again, our measure underestimates the real increase in H3 mRNA, since 28S rRNA increases when ESCs differentiate (Sampath et al., 2008).

Next, we measured the amount of H3 protein relative to total DNA content (Figure 3D): 8-day EB cells contain on average 2-fold more H3 histone than their ESC counterparts (Figure 3E; *p* = 0.0021; Kruskal-Wallis test).

### ESCs express less H3 mRNA and contain less H3 histone compared to differentiated neuronal and endodermal cells

**Figure 4:**
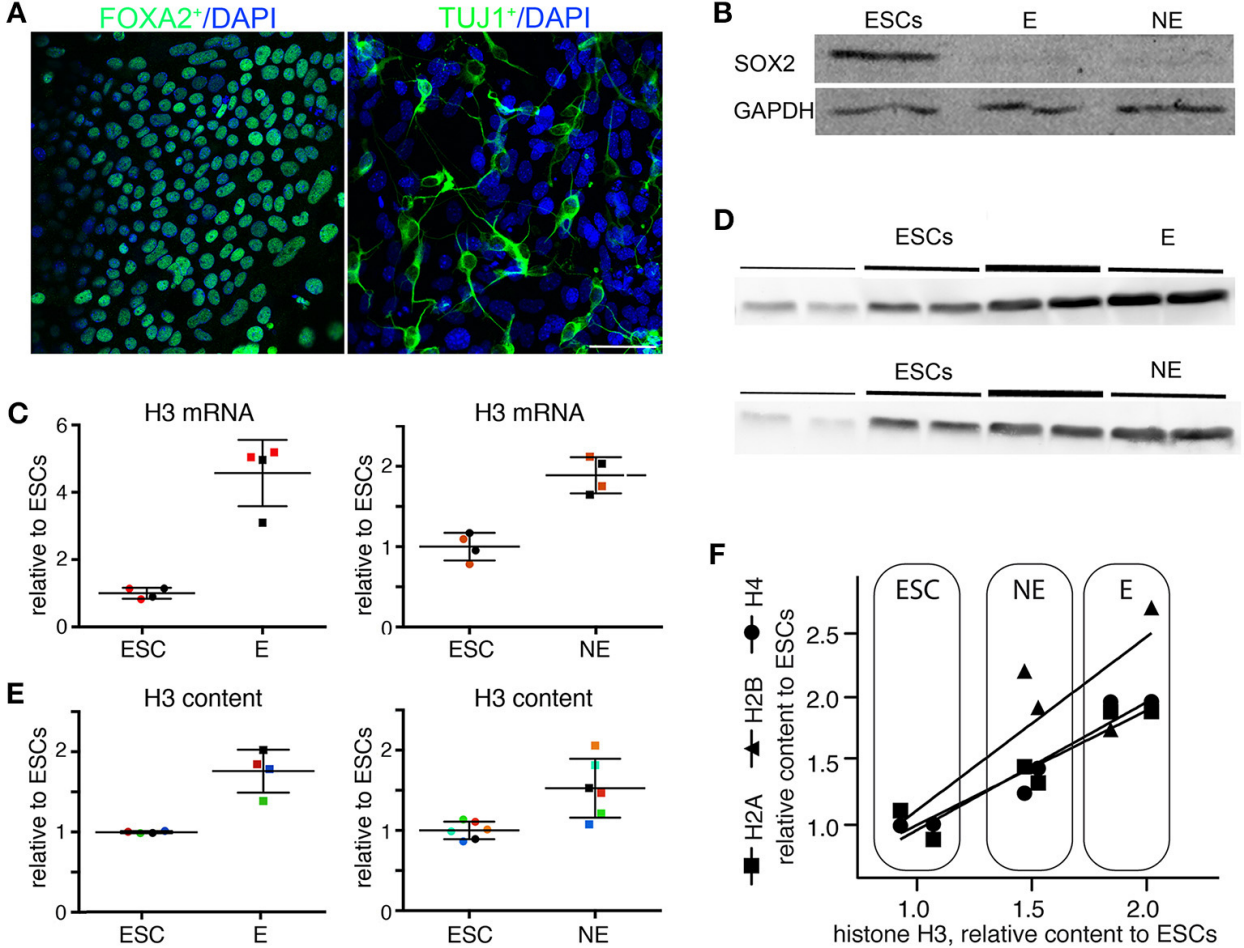
**(A)** R1 and CCE ESCs were specifically differentiated to TUJ1 expressing neuronal cells (NE) or to FOXA2 expressing endodermal cells (E). We found no statistical difference between R1 and CCE cells, between NE cells or E cells derived from the different ESC lines. Immunofluorescence for TUJ1 and FOXA2. Scale bar 50 μm. **(B)** ESCs express SOX2, whereas ESC-derived endodermal and neural cells do not. **(C)** ESCs express less H3 mRNA than differentiated E and NE cells. Each dot represents a different biological replicate. Color codes identify samples analyzed in the same batch; different colors represent samples analyzed in different days. The mean and standard deviation are indicated by horizontal lines. The groups are statistically different (Mann–Whitney test, *p* = 0.029). **(D)** Western blot for H3 histone. Lines of different thickness represent loading amounts: equivalent to 100, 200, 300 ng of DNA. **(E)** Endodermal (E) and neuronal (NE) cells contain more H3 protein than ESCs. The different samples are normalized for H3/DNA ratio. The values are normalized relative to the mean of the ESC group, which is set to 1. Each dot represents a different biological replicate. Color codes identify samples analyzed in the same batch; different colors represent samples analyzed in different days. The groups are statistically different (Mann–Whitney test, *p* = 0.029 for ESCs vs. E and *p* = 0.0087 for ESCs vs. NE). **(F)** The levels of all core histones increase coordinately in NE and E cells differentiated from ESCs. Two biological replicates from the same set of samples analyzed in **(C)** were analyzed for the levels of H2A, H2B, and H4 levels. The results are represented as linear regression between the level of H3 and the level of other core histones. The levels of each core histone correlate with the levels of histone H3 (Pearson *r*^2^: H2A = 0.87, *p* = 0.0062; H2B = 0.72 and *p* = 0.032; H4 = 0.93 and *p* = 0.002). The results are compatible with a 1:1 stoichiometric ratio of each histone to each other, as expected.

ESCs were then specifically differentiated toward the neuronal and the endodermal lineage using lineage specific inducing factors bFGF and A-activin, respectively (Li et al., 1998; Kubo et al., 2004). At the end of differentiation neuronal cells (NE) express the surface marker TUJ1 and endodermal cells (E) express the transcription factor FOXA2, an endodermal marker (Figure 4A). Both proteins are absent from ESCs. SOX2 is present ESCs but not in NE or E cells (Figure 4B). The absence of SOX2 further suggests that NE cells have already exited the cell cycle.

ESCs, NE and E cells were lysed and analyzed as indicated before; each sample is a biological replicate. The expression of H3 mRNA relative to 28S increases in NE and E cells relative to ESCs (*p* = 0.029, Mann–Whitney test), and becomes 2-fold larger on average in NE cells and 4-fold larger on average in E cells (Figure 4C).

Both E and NE cells contain on average 1.7-fold more H3 histone than their ESC counterparts (Figures 4D,E; *p* = 0.0286 for E and *p* = 0.0087 for NE; Mann–Whitney test).

We also analyzed two biological replicates from the set of samples obtained by directed differentiation of ESC to NE and E cells for the content of histones H2A, H2B, and H4 (Figure 4F). The content of each histone correlated with the measured amount of histone H3 (*n* = 6 for each histone; correlations: H2A *r*^2^ = 0.87 and *p* = 0.0062; H2B *r*^2^ = 0.72 and *p* = 0.032; H4 *r*^2^ = 0.93 and *p* = 0.002). Indeed, as expected the measured levels of each histone were statistically compatible with equal stoichiometry (H2A 0.9: H2B 1.3: H3 1.0: H4 1.0), indicating that the level of histones grows coordinately during directed differentiation.

## Discussion

**Figure 5:**
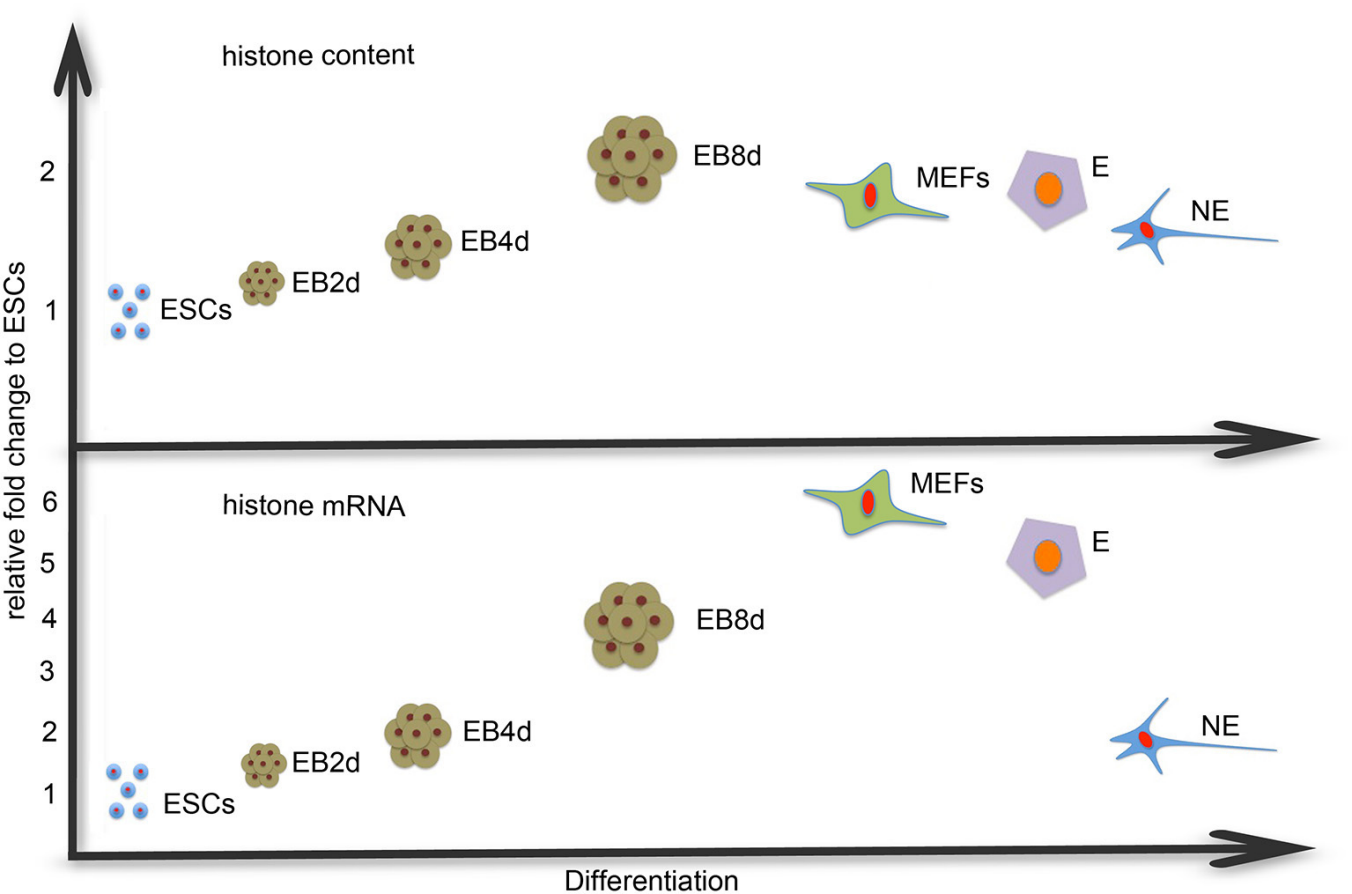
**H3 mRNA and protein content vs. differentiation**. ESCs express and contain less histone H3 than the differentiated cells. The difference is larger in mRNA levels than in the protein content and there is variability between the various differentiated cell types.

Our group recently showed that the amount of core histone proteins, and thus of nucleosomes, is not rigidly determined by the amount of DNA to be packaged: in cells lacking the HMGB1 protein, which can bend DNA and facilitate histone deposition, histone levels are reduced by more than 20%, and the level of total RNA (including mRNAs) is increased by more than 30% (Celona et al., 2011). While these data confirm that DNA packaging into nucleosomes restricts transcription, we now have considered the reverse proposition—whether cells with high levels of transcription and high chromatin accessibility contain less histones. We show here that ESCs, pluripotent cells that correspond to the *in vivo* developmental stage of inner mass cells in the blastocyst (E 3.5) and are prototypes of cells with high chromatin accessibility (Albert and Peters, 2009), express less H3 mRNA and contain less H3 protein than differentiated cells—MEFs, endodermal and neuronal cells (Figure 5). Moreover, the level of histone mRNA and protein increases during spontaneous differentiation from ESCs to EBs and directed differentiation to neuronal and endodermal cells. Our claim is based on the careful measure of H3 mRNA and protein levels, and is confirmed by more limited yet statistically significant data on the other core histones H2A, H2B, and H4.

mRNA levels can be accurately determined by qPCR, if care is taken to use primers that can amplify transcripts from the more than one dozen histone H3 genes in the mouse genome. However, the measured amount must be normalized against another RNA molecule, and we chose the 28S rDNA molecule. This is not ideal, since ribosomes increase in number when ESCs differentiate (Sampath et al., 2008), but this bias can only lead to an overestimation of the fraction of histone H3 mRNA molecules in ESCs relative to differentiated cells. Therefore, differentiated cells contain *at least* between 2-fold and 10-fold more histone H3 mRNA than ESCs. This indicates that histone H3 transcripts vary much more sharply than histone H3 protein during ESC differentiation. While not entirely expected, our finding is in agreement with the observation that in ES cells mRNA abundance does not reflect the amount of protein synthesized at any time (Lu et al., 2009).

Histone protein levels are more difficult to establish accurately, since the difference sought is necessarily limited, in the order of 20–30%. More precisely, what we measure is the quantity of H3 histone relative to DNA, a measure of nucleosome density (as more than 99.5% of histones are assembled within nucleosomes). To this end, we accurately determine the amount of DNA within a sample of lysed cells, load the equivalent of the specified amount of DNA on a SDS-PAGE gel, and perform a Western blot that recognizes total H3 histone, irrespective of modifications. We showed previously that the accuracy of Western blots is within 10%, as confirmed by SILAC data (Celona et al., 2011). Our measurements are carried out on several (usually 6) technical replicates and many biological replicates from experiments performed in different days. Non-parametric statistical tests avoid assumptions about measurement distributions. These rigorous analyses converge on indicating that MEFs, neuronal and endodermal cells all contain more H3 histone than ESCs, typically about 50% more. Conversely, this indicates that ESCs contain about 30% less histone H3 than different types of differentiated cells. This is not to say that all differentiating or differentiated cells will contain the same amount of DNA; in fact, cells in 8-day EBs appear to contain even more H3 histone. As expected, the levels of the other core histones appear to vary coordinately with that of H3.

We previously measured a similar 30% reduction in core histone levels in *Hmgb*1^−/−^ MEFs relative to wt MEFs (Celona et al., 2011), and a similar reduction in core histone levels was found in senescent mammalian cells (O'Sullivan et al., 2010). We also showed recently that macrophages exposed to LPS reduce their content of core histones within 4 h, and then resynthetize them (De Toma et al., 2014). There is no obvious single common denominator among these conditions, and in particular stem cells and senescent cells are at opposite extremes of the spectrum in developmental capacities. In senescent cells, reduced histone biosynthesis was attributed to persistent DNA damage (O'Sullivan et al., 2010). In contrast, ESCs repair DNA more efficiently than differentiated cells on one hand (Maynard et al., 2008), and on the other hand are particularly prone to undergo apoptosis following DNA damage (Aladjem et al., 1998); both mechanisms would reduce unrepaired DNA damage in the ESC pool. We therefore suggest the biological mechanisms underpinning reduced histone content might be different in different cells and in different conditions.

It is also notable that when histones levels are reduced, they always appear be reduced by approximately 30%. While it is entirely possible that this is a mere coincidence, it is also possible that *Hmgb*1^−/−^ MEFs, freshly activated macrophages, senescent cells, and ESCs contain close to the minimum amount of histones and nucleosomes compatible with sufficient packaging of chromatin—in other words, that we measure similar amounts of H3 histone because a lower amount would be incompatible with survival.

Based on our previous experience on *Hmgb*1^−/−^ MEFs, a reduced histone (and nucleosome) content should make DNA more accessible to both transcription and damage.

*Hmgb*1^−/−^ cells have a genomewide increase in RNA levels of about 30%, which is fully compatible with current mechanisms of how polymerase II contends with overcoming nucleosomes along the. ESCs are generally considered to transcribe a large diversity of genes, some of which are shut down as differentiation progresses. However, transcribing more genes than differentiated cells is conceptually different from transcribing each gene a bit more or a bit faster. To our knowledge, comparisons of the speed of polymerase II on model genes in ESCs and differentiated cells have not been performed.

*Hmgb*1^−/−^ cells are also more accessible to DNA damage: a given amount of UV irradiation produces about double the amount of thymine dimers relative to wt cells (Giavara et al., 2005), and gamma rays about double the amount of double strand breaks (Celona et al., 2011). However, such DNA damage is also repaired more efficiently (Giavara et al., 2005; Celona et al., 2011). As noted before, unirradiated ESCs do not harbor more DNA damage than differentiated cells, but to our knowledge the amount of DNA damage upon irradiation has not been specifically tested. It is known, however, that ESCs do repair damaged DNA more effectively than differentiated cells (Maynard et al., 2008).

A reduced histone content causes a decrease in the number of nucleosomes, and hence of the physical substrate over which to appose histone marks. Transcription of a very large number of genes in ESC has so far been attributed to the presence of posttranslational modifications on core histones that increase accessibility and to the relative paucity of heterochromatic posttranscriptional modifications (Albert and Peters, 2009). Recent analyses found that the fraction of the genome enriched in H3K9me2, a heterochromatic mark, is not different in ESCs and differentiated cells (Filion and van Steensel, 2010; Lienert et al., 2011), but the interconnection between histone content and histone modification has not been explored.

A reduced number of nucleosomes should also affect the activity of chromatin remodeling complexes, including PBAF that appears to control Nanog expression by modulating chromatin structure (Schaniel et al., 2009).

Since the variation in such a fundamental feature as the number of nucleosomes is bound to have deep and wide-ranging effects on chromatin organization, we expect that a low histone content would be integral to maintaining the ES cell identity, and thus for totipotency and stemness.

### Conflict of interest statement

The authors declare that the research was conducted in the absence of any commercial or financial relationships that could be construed as a potential conflict of interest.
